# Implementation of a Novel Nanobody Panel for the Efficient Capture of Extracellular Vesicles from Human Plasma

**DOI:** 10.3390/molecules30183677

**Published:** 2025-09-10

**Authors:** Marija Tursunović, Lidija Filipović, Ninoslav Mitić, Sanja Stevanović, Milica Spasojević Savković, Ario de Marco, Milica Popović

**Affiliations:** 1 Innovative Centre of the Faculty of Chemistry, 11158 Belgrade, Serbia; mtursunovic@chem.bg.ac.rs (M.T.); lfilipovic@chem.bg.ac.rs (L.F.); smilica84@gmail.com (M.S.S.); 2 Institute for the Application of Nuclear Energy, INEP, University of Belgrade, 11080 Belgrade, Serbia; ninoslavm@inep.co.rs; 3 Institute for Chemistry, Technology, and Metallurgy, University of Belgrade, Njegoševa 12, 11000 Belgrade, Serbia; sanjas@ihtm.bg.ac.rs; 4 Laboratory for Environmental and Life Sciences, University of Nova Gorica, 5000 Nova Gorica, Slovenia; ario.demarco@ung.si; 5 Faculty of Chemistry, University of Belgrade, Studentski trg 12-16, 11158 Belgrade, Serbia

**Keywords:** extracellular vesicles, nanobodies, bioinformatics, immunoaffinity chromatography

## Abstract

Extracellular vesicles (EVs) are nanoscale particles released by cells and are significant components in intercellular communication. Their ability to reflect the molecular state of parental cells and their presence in body fluids make them increasingly recognized as promising non-invasive biomarkers for different pathological conditions. However, the existence of different EV populations and frequent co-isolation of contaminants present challenges for EV purification and downstream analyses. In this study, we used three novel nanobodies (VHH) for selective isolation of EVs from human plasma. Nanobodies were obtained by direct panning on EVs. All examined nanobodies have excellent physicochemical properties resulting in excellent expression and solubility. The three nanobodies being studied—NA8, ND10_1_, and ND10_2_—share a conserved VHH scaffold but exhibit different loop architectures. The Biopython ProtParam module was used for calculation of VHH physicochemical properties, while sequence alignments for evaluation of variations were performed with the Biopython pairwise2 module. In addition, structural modeling of nanobodies with AlphaFold revealed notable differences in CDR3 conformations. VHH were produced in *E. coli*, and upon immobilization onto a solid carrier, they were used for immunoaffinity-based capture of EVs from human plasma. Combined characterization of isolated EVs supports efficient application of an immunoaffinity-based system based on such nanobodies for the isolation of EVs from human plasma to be used for downstream analyses.

## 1. Introduction

All cells release EVs, nanoscale particles surrounded by a lipid membrane, into the extracellular environment. EVs transport a variety of bioactive molecules to local and distant cells, thereby altering their function [1]. They are often classified based on their size, biogenesis, and cargo composition as exosomes (EXO), microvesicles (ectosomes), and apoptotic bodies [2]. Apoptotic bodies are formed during the process of programmed cell death and are the largest in size [3]. Unlike microvesicles, which originate from outward budding off the plasma membrane [4], exosomes, whose diameter usually ranges from 30 to 200 nm, are formed through fusion of multivesicular bodies (MVBs) with the plasma membrane, and their diameter usually ranges from 30 to 200 nm [5,6]. EVs have a significant role in intercellular communication by transporting bioactive molecules, including proteins, lipids, and nucleic acids between cells [7]. Thus, EVs, in particular exosomes, contribute to the modulation of numerous physiological and pathological processes; for instance, immune response regulation, tissue repair, and tumor progression [8,9,10]. Their presence in different biological fluids [11,12,13], as well as their ability to reflect the molecular composition of parent cells, makes them recognized as a non-invasive diagnostic tool [14]. Furthermore, exosomes are also considered as a promising drug delivery system due to their low immunogenicity, capability of crossing biological barriers, and ability to release their cargo directly into the target cell cytoplasm [15]. In addition, the presence of specific patterns of displayed proteins can be exploited to obtain targeted delivery of EXO to specific classes of cells [16].

Given the important roles of EVs in diagnostics and therapy, it is crucial to establish efficient and reproducible isolation methods that would preserve their integrity and result in high yield and purity. Common methods used for EVs isolation, such as differential ultracentrifugation, size-exclusion chromatography (SEC), and precipitation-based techniques, separate EVs from biological fluids based on their size or density. However, these techniques are time-consuming and often result in EVs that are co-isolated with other particles of similar physicochemical properties [17]. On the contrary, immunoaffinity-based techniques provide a higher degree of specificity by allowing for the selective capture of EVs based on their surface markers. Commercial IgG antibodies specific for EV surface proteins, such as tetraspanins (CD9, CD63, CD81), resulted in successful purification of EVs [18,19,20]. This approach has the potential to selectively capture EVs of different origin according to their specific combination of surface markers but the high costs of conventional antibody production still represent a substantial limitation to their broader application [21].

To overcome such a bottleneck, single-domain antibodies (nanobodies or VHH) are emerging as a promising alternative. VHHs are single-domain antibody fragments derived from *Camelidae* heavy-chain only IgG antibodies and show desirable properties, such as thermal resistance, high solubility, and remarkable resistance to pH changes [22,23,24,25,26]. In addition, they can easily be produced in bacteria and yeast cells, which substantially decreases the price of their production [26]. The unique monomeric structure of VHHs allows for multimerization, which can lead to increased avidity and binding to multiple antigens [27]. VHH-based capture of EVs offers distinct mechanistic advantages over traditional antibody-based methods. Nanobodies small mass (~15 kDa) allows obtaining high density when used to activate surfaces. Furthermore, VHHs exhibit superior stability and the ability to access epitopes inaccessible to conventional IgG antibodies (~150 kDa). Therefore, they are particularly suitable for EV immunocapture using beads or chromatographic matrixes. At the same time, these properties enable displayed VHHs to serve as efficient targeting moieties for EV-based drug delivery systems, facilitating precise therapeutic applications [28]. Moreover, the selection of new binders can be accomplished via phage display techniques [29], even directly against complex targets, such as whole EVs [30], without prior identification of specific surface markers.

In our previous studies, we successfully implemented a method for EVs isolation from various sources based on a set of five different nanobodies [31,32]. In the current work, this approach is advanced by introducing a new set of nanobodies, selected from a naïve library by direct panning on EVs from human breast cancer cell line (SKBR3). Furthermore, the potential of each individual VHH clone for selective and efficient capture of EVs from human plasma was evaluated.

## 2. Results

### 2.1. Selection of Anti-EVs VHH

**Figure 1:**
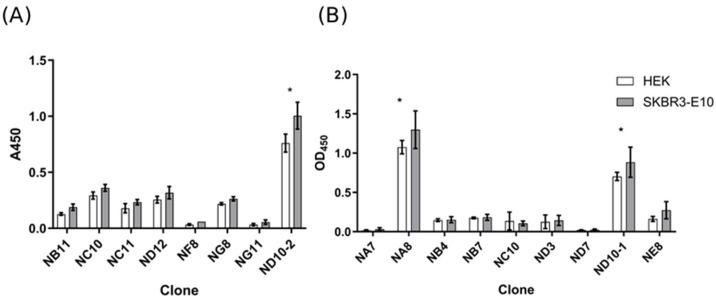
Phage-ELISA of clones selected by panning was performed to confirm their specific binding to EV-enriched fractions recovered from SKBR3 and HEK293 cells. Assay microplates were coated with EV-enriched samples (0.1 μg/well of protein) and bound phages were detected with HRP-labeled anti-M13 antibodies. An irrelevant phage-displayed nanobody was used as a negative control. (**A**) Results from the first round of screening. (**B**) Results from a successive second screening round. The error bars indicate standard deviations for triplicate measurements. * Denotes significant different values (Tukey’s HSD, *p* ≤ 0.05).

An in vitro selection strategy was adapted for isolating nanobody binders against EVs. The workflow involved a differential panning procedure, where EV-enriched fractions from HEK293 cells were used in the depletion step, and SKBR3-derived EVs were used in the enrichment step. EVs were stabilized by binding them to magnetic beads decorated with anti-EVs-VHHs. The use of such reagents facilitated the efficient washing and recovery of bound phages. After two rounds of selection, the recovered clones were screened by phage ELISA against EVs from both cell types. A subset of EV-reactive clones was selected for sequencing, revealing several unique nanobody sequences. Despite the depletion step used to remove binders recognizing common epitopes, the selected binders did not show sufficient discriminatory properties and rather recognized conserved EV epitopes (Figure 1). This result suggests that the library dimension was too elevated for a complete depletion with the amount of EVs we used. Direct panning on EVs has been performed only occasionally [30], and never using a depletion step; therefore, the collected data represent an important knowledge advancement showing that the used protocol, derived from that used for panning on whole cells, cannot be directly applied to EVs. Given their smaller dimension, the number of displayed antigens is relatively low and, therefore, effective depletion would probably require extremely more EVs than we used.

### 2.2. Physicochemical Analysis of VHH Nanobodies and Sequence Comparison

**Figure 2:**
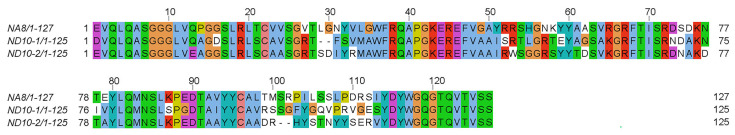
Multiple sequence alignment for protein variants NA8_,_ ND10_1_, and ND10_2_. The alignment shows residue matches. The residues are color-coded, commonly using ClustalX coloring, which groups amino acids by chemical properties: Blue—hydrophobic; Red—acidic; Magenta—basic; Green—polar uncharged; Orange/Yellow—special cases.

The three nanobodies had molecular weights ranging from 13.5 to 14.0 kDa and isoelectric points in basic pH (pI value: 8.59–9.30), suitable for optimal solubility at the physiological pH. The highest molecular weight was found for NA8, due to its longer CDR1. An instability index below 40 predicted in vitro stability of ND10_2_ (Supplementary Table S1). Calculated GRAVY values were negative for all the nanobodies, reflecting a hydrophilic nature, with ND10_2_ being the most hydrophilic (−0.632), while ND10_1_ (−0.241) and NA8 (−0.393) were less hydrophilic (Supplementary Table S1). The aliphatic index ranged between 55.52 and 73.62, the highest occurring in NA8, suggesting greater thermal stability. Aromaticity values were comparable (~0.11). Sequences of nanobodies are shown in Figure 2.

VHH sequences display canonical framework (FR1, FR2, FR3, and FR4) and CRD loop (CDR1, CDR2 and CDR3) sequence regions. The framework regions (FR1, FR2, FR3, and FR4) exhibit a high degree of conservation across all three variants. In contrast, the CDRs, particularly CDR3 (residues 104–116), shows higher degree of variability, which potentially reflects their critical function in antigen recognition and specificity.

The amino acid sequences of the VHH three complementarity-determining regions (CDRs) are given in Table 1.

To analyze structural diversity and conservation of nanobody sequences, lengths of CDR1–3 were compared (Table 1). The three VHH sequences had CDRs that differed in composition and length. CDR1 had six to eight residues, the longest of which was from NA8. The CDR2 sequences were as well short in the three nanobodies, having seven to eight residues each. The CDR3 regions had the greatest variation in terms of length, with NA8 and ND10_1_ having longer loops (20 residues) and ND10_2_ with shorter loops (18 residues). It is interesting that all CDR3 sequences contained the conserved terminal motif “YDY,” which is in accordance with known VHH characteristics [27].

### 2.3. Structural Interpretation of NA8, ND10_1_, and ND10_2_

**Figure 3:**
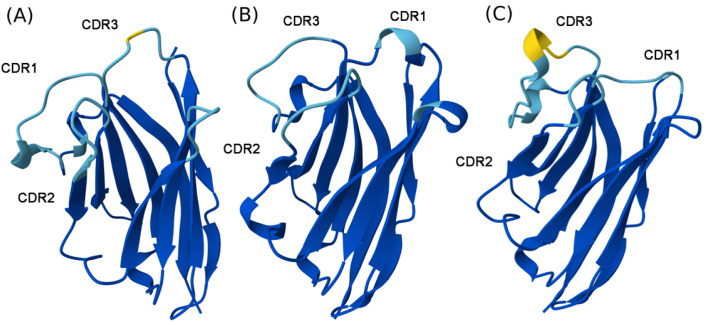
Predicted structures of nanobodies NA8 (**A**), ND10_1_ (**B**), and ND10_2_ (**C**) generated using AlphaFold Server. View is oriented to show side-chain variability across framework; pIDDT scores are color coded: Very high (plDDT > 90)—blue; Confident (90 > plDDT > 70)—light blue; Low (70 > plDDT > 50)—yellow.

In silico predictions of nanobodies was performed using AlphaFold Server [33]. All the predicted structures exhibited the characteristic immunoglobulin fold with a β-sheet scaffold forming the core of the domain. The complementarity-determining regions CDR1, CDR2, and CDR3 were resolved well in all of them. The framework regions were predicted with high confidence and were found to be predominantly colored dark blue, indicated by high pLDDT scores (Figure 3). In contrast, the CDR loops, and especially CDR3, were more structurally diverse and less confidently predicted, with regions shown in pale colors like yellow, reflecting lower model confidence. The single model ND10_2_ contained a helical turn within the CDR3 loop, differing from the longer loops in nanobodies NA8 and ND10_1_. The model accuracy was assessed by applying MolProbity scores that predict reliable results for values below 1.5 are usually considered as hallmarks of high structure accuracy [34]. In our case, all the scores fell below the 1.5 threshold (1.34, 1.11 and 1.50 for NA8, ND10_1_ and ND10_2,_ respectively).

### 2.4. Purification of Nanobodies

**Figure 4:**
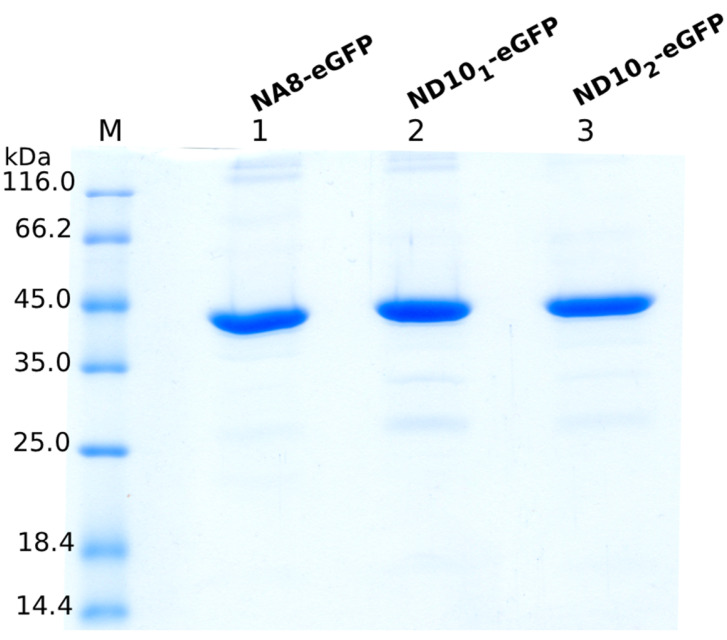
SDS-PAGE analysis of purified VHH-eGFP constructs. Three different constructs (NA8-eGFP, ND10_1_-eGFP, and ND10_2_-eGFP) were expressed in *E. coli* and purified. Bands at ~45 kDa correspond to the expected molecular weight of the nanobody fused to enhanced green fluorescent protein.

Small-scale expression conditions were assessed prior to VHH purification to produce them in soluble form. VHH-eGFP constructs were expressed in *E. coli* cells and then purified using IMAC. SDS-PAGE analysis (Figure 4) showed clear bands that corresponded to the expected molecular weight (~45 kDa) of the VHH-eGFP constructs, confirming successful purification and their high purity. The constructs were produced in yields of 14–32 milligrams per liter of culture media.

### 2.5. Size Distribution of EVs Isolated Using Nanobody-Based Approach

**Figure 5:**
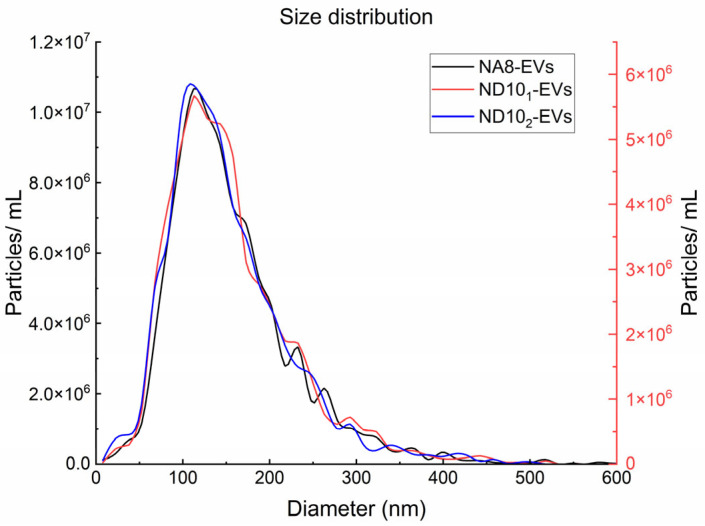
Size distribution of EVs isolates. Dual y axis representation indicates that all EV isolates exhibit similar size distributions with the expected range of EVs. ND10_1_-EVs size distribution is shown on secondary y axis.

Produced VHH constructs were immobilized upon purification onto a solid methacrylate-based carrier and used for EVs purification. Nanoparticle tracking analysis (NTA) was used to determine the concentration and size of EVs isolated from human plasma. Quantitative data (Table 2) indicate that the highest yield (4.57 × 10^9^ particles) and a median diameter of 138.1 ± 2 nm were obtained, when NA8 was used for EV capture. ND10_2_-EVs exhibited a similar profile with a slightly lower yield (2.37 × 10^9^ particles) and a median diameter of 134.6 ± 1 nm. In contrast, ND10_1_-EVs showed lower yield (1.27 × 10^9^ particles) and a comparable median diameter (137.3 ± 3 nm). All three EV isolates displayed similar size distribution profiles, with the majority of particles ranging from ~50 nm to ~300 nm and the peak around 100 nm. (Figure 5).

### 2.6. Protein Profile of Isolated EVs and Detection of Surface Markers

#### 2.6.1. SDS-PAGE Analysis

**Figure 6:**
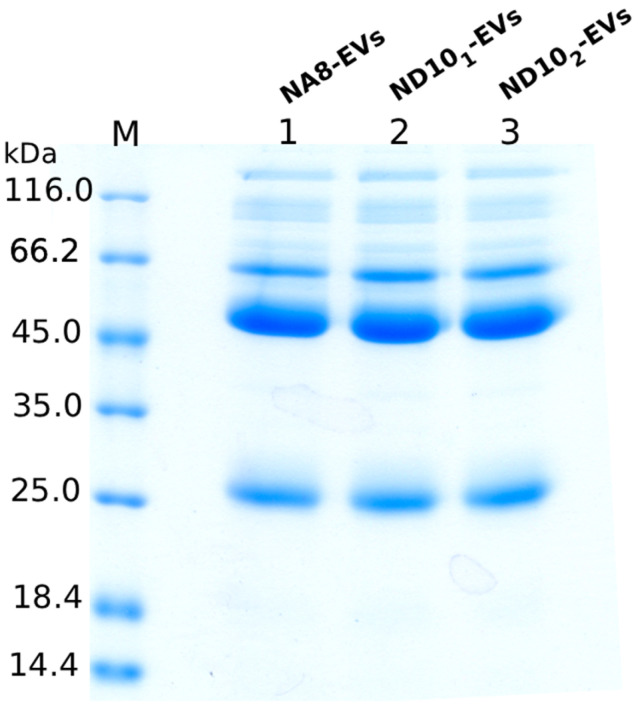
SDS-PAGE protein profiles of isolated EV. Equal volumes (25 uL) of each EV isolate were loaded into the wells.

To evaluate the protein pattern of EV isolated by our immobilized nanobodies, SDS-PAGE was performed. Upon elution, an equal volume of each EV sample was loaded and separated on a 12% gel in reducing conditions. (Figure 6; Supplementary Figure S1).

The vesicles display similar protein bands, with the most prominent ones at ~25 kDa, as well as between ~45 and ~66 kDa, indicating that similar EV subpopulations were captured.

#### 2.6.2. Detection of Surface Markers with Flow Cytometry

**Figure 7:**
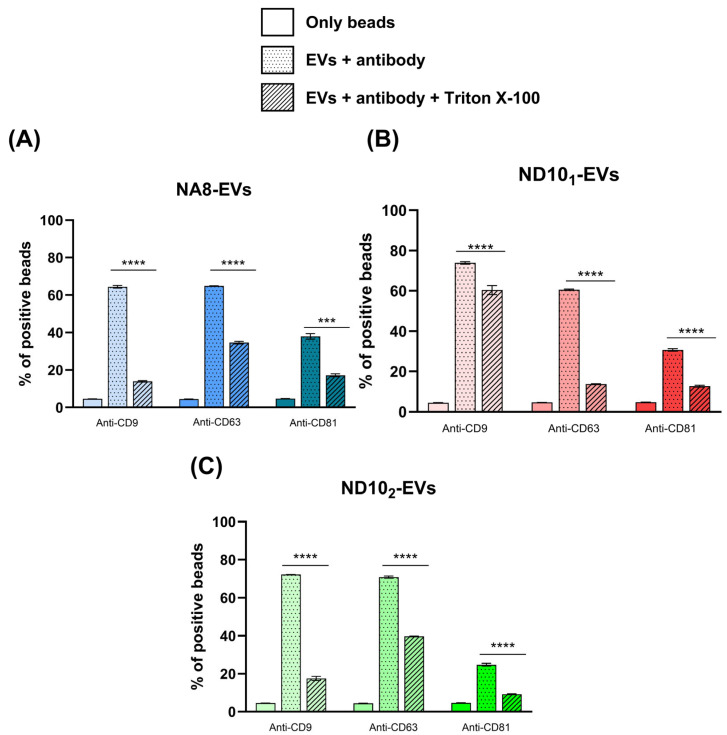
Detection of surface markers in EVs isolated with NA8 (**A**), ND10_1_ (**B**), and ND10_2_ (**C**). Beads alone were used as a negative control. Dotted histograms in all samples represent detection levels of surface markers obtained with anti-CD9, anti-CD63, and anti-CD81 antibodies, while the results obtained after treatment of vesicles with detergent is represented in histograms with lines. A significance level of *p* ≤ 0.05 was used for analysis of variance, implemented using the one-way ANOVA test followed by Tukey’s post hoc test (*p* ≤ 0.05). Values with significance level of *p* ≤ 0.05 were considered statistically different. *** Denotes *p* value of 0.0005. **** Denotes *p* values less than 0.0001.

Flow cytometry analysis was performed by immobilizing EVs onto latex beads and using three different fluorescently labeled monoclonal antibodies against the standard EV biomarkers CD9, CD63, and CD81. All isolates displayed a positive shift in the fluorescence intensity in comparison to autofluorescence of the beads themselves (Figure 7). Positive populations of beads were detected in NA8-, ND10_1_-, and ND10_2_-isolated EVs: CD9 (64.40%, 60.55%, 72.50%), CD63 (64.85%, 64.85%, 70.90%), and CD81 (37.95%, 30.65%, 24.80%), respectively. To verify that positive signals originate from intact vesicles and not protein aggregates, a detergent control with Triton X-100 was included. Treatment of EVs with detergent significantly reduced the fluorescence signal, indicating the presence of lipid membrane structures.

### 2.7. Evaluation of Protein and Lipid Content in EVs Isolates

#### 2.7.1. Quantification of Proteins and Lipids with Colorimetric Assays

The Bradford assay and sulfo-phospho-vanillin (SPV) assay were used for quantification of proteins and lipids in isolates, respectively. The ND10_1_-EV exhibited the highest protein yield, which was significantly higher than those of both NA8-EV and ND10_2_-EV. Among three isolates, ND10_2_-EV showed the highest lipid yield, although no statistically significant differences were observed in lipid content across all groups. (Table 3). Calculated P/L ratios were 2.740 ± 0.074, 4.248 ± 0.323 and 2.536 ± 0.153 for NA8, ND10_1_, and ND10_2_, respectively, indicating satisfactory level of purity for plasma samples [35].

#### 2.7.2. Attenuated Total Reflectance Fourier-Transform Infrared Spectroscopy (ATR-FTIR)

**Figure 8:**
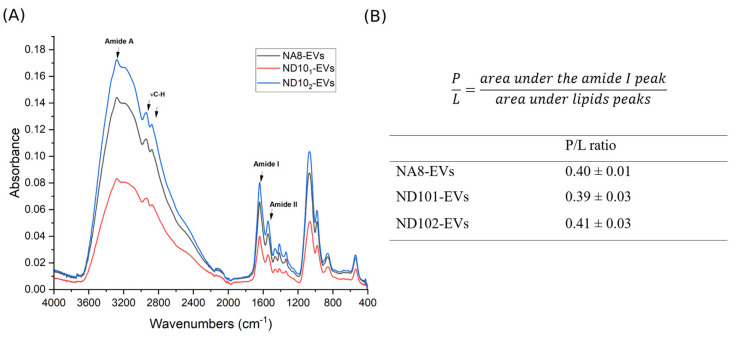
ATR-FTIR spectra of EVs isolated with NA8, ND10_1_ and ND10_2_ nanobodies (**A**) and P/L ratios for EVs calculated from the spectra (**B**).

To evaluate the biochemical composition of the isolated extracellular vesicles, ATR-FTIR spectroscopy of all EV isolates was performed. The spectra display consistent profiles across all samples with prominent bands corresponding to amide I (~1640 cm^−1^) and amide II (~1540 cm^−1^), originating from C=O stretching vibrations and N-H bending vibrations of the peptide bonds, respectively [36]. In addition, the band labeled as amide A at 3280 cm^−1^ originates from N-H stretching vibrations of the peptide groups in proteins [36]. Prominent absorption bands in the region ~2985–2835 cm^−1^ correspond to symmetric and asymmetric CH_2_ stretching vibrations from the lipid acyl chain, confirming presence of the lipid bilayer [37]. Upon integrating an area under the curve in the range of 2985 to 2840 cm^−1^ originating from lipid bands and amide I area (1725–1575 cm^−1^), protein-to-lipid ratios (P/L) in all samples (Figure 8B) were calculated. The determined P/L ratios were 0.40, 0.39 and 0.41 for NA8, ND10_1_ and ND10_2_, respectively.

### 2.8. Morphology Evaluation

**Figure 9:**
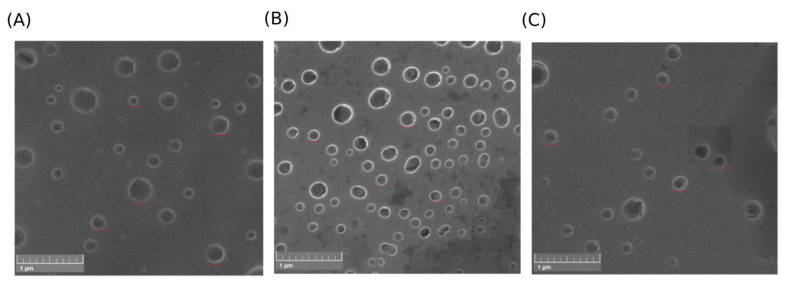
SEM analysis of EVs isolated with affinity-approach based on NA8 (**A**), ND10_1_ (**B**), and ND10_2_ (**C**) constructs. The red lines denote representative EVs in isolates.

**Figure 10:**
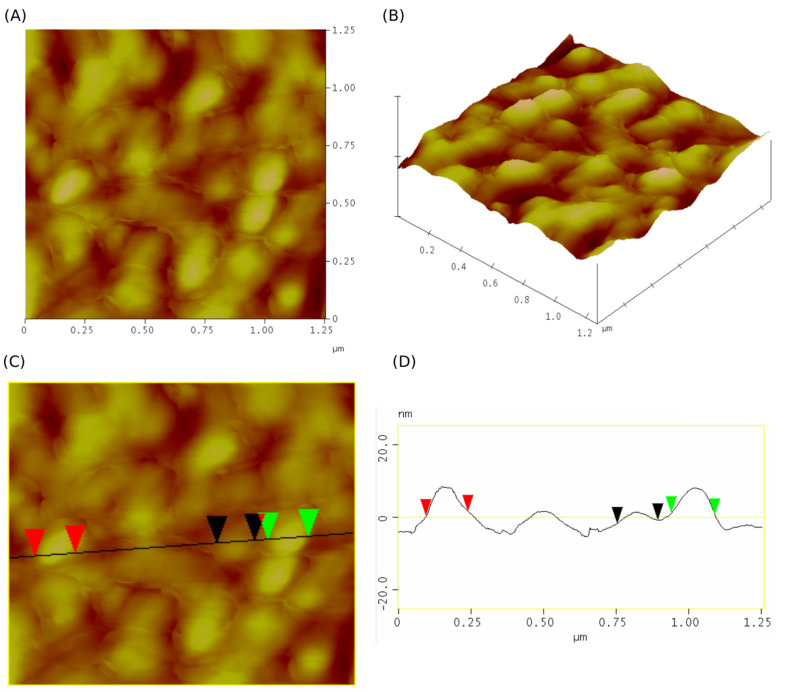
AFM analysis of EVs isolated with ND10_1_ (**A**,**B**) represent two-dimensional and three-dimensional analyses. (**C**) represents a cross-sectional profile analysis. (**D**) represents a linear profile with markers.

Scanning electron microscopy (SEM) and atomic force microscopy (AFM) were used for determination of morphology and structural integrity of EVs isolated with the nanobody-affinity approach. Obtained pictures from both SEM (Figure 9) and AFM (Figure 10) indicated a high abundance of EVs in samples, confirming successful isolation and purification. Notably, the EVs showed a characteristic shape and intact morphology, indicating that upon elution, well-preserved vesicles were obtained.

The SEM (Figure 9) images revealed a heterogeneous population of EVs with predominantly spherical morphology and diameters ranging from approximately 50 to 200 nm, which is consistent with the size profiles obtained with NTA. Vesicles were generally well-preserved with smooth surfaces. Minimal background debris were observed across all panels, suggesting effective purification. The presence of clearly defined vesicle boundaries and uniform morphology supports the successful isolation and structural integrity of the EVs.

Atomic force microscopy (AFM) was employed to image the surface morphology and topography of isolated ND10_1_-EVs in panels A, B, and C. Two-dimensional and 3D AFM images displayed (Figure 10) the typical spherical shape of EVs. The diameters of these globules ranged from 139 to 150 nm (red markers on the profile line denote globules of 144.47 nm in diameter, while green markers and black markers denote globules of 149.36 nm and 139.57 nm in diameter, respectively).

## 3. Discussion

Liquid biopsy procedures based on readily available biomarkers play an important role in early diagnosis of various pathological conditions. EVs are at the forefront as a potential source of the next readily available and affordable biomarker, especially in oncology [38]. Plasma represents a substantial and readily available source of EVs, thus serving as an optimal source for liquid biopsy applications, especially in larger screening campaigns [39]. However, isolating plasma EVs is often challenging due to the presence of plasma proteins and lipoproteins [40,41]. Therefore, implementation of methods that would ensure high-specificity EV capture, such as immunoaffinity approaches, may allow for overcoming these limitations. Antibodies specific for different EV markers, including EpCAM, MHC class II, or different CD molecules, have been successfully used for EVs isolation [20,42,43,44]. However due to high costs of their production, building a screening system based on monoclonal antibodies remain expensive processes and pose a barrier to the widespread usage.

Nanobodies can have very diverse paratope structures, which can recognize a large number of epitopes, especially hidden or recessed epitopes [45,46]. Genetic engineering, site-directed mutagenesis and in silico design tools can be applied to tune the nanobody structure with the aim of improving their binding affinity and specificity to a great extent [47,48,49]. The development of naïve, synthetic, and semisynthetic VHH libraries can offer a great platform for introduction of new binders [50,51,52]. Furthermore, panning techniques allow for identification of nanobodies for recognition of selective EV surface markers, e.g., for identifying nanobodies that show higher affinity towards cancer cell vesicles, in comparison to vesicles derived from healthy cells [30]. EVs can be instable during panning conditions and this can result in loss of auspicious binders. Stabilization of EVs on epoxy beads or microplate wells by means of VHH-dependent immunocapture leads to increased stability of the material and consequently less material needs to be used for screening procedures The lack of ability to select VHHs that specifically recognize cancer-derived EVs might stem from the lack of truly tumor-exclusive surface antigens or by their limited expression with respect to common tetraspanins such as CD9, CD63, and CD81, which are present on both normal and malignant cells, making selective targeting difficult [53]. A more aggressive depletion step will be assessed in the future with the aim of better saturating the library and isolating the binders specific for the rare selective biomarkers. Additionally, many putative tumor-associated proteins are intracellular or shed in soluble form [54], reducing their accessibility on the vesicle surface for nanobody binding. Overcoming these obstacles will likely require advanced single-EV sorting technologies. Through this targeted approach, new precise diagnostic tools may be developed for recognition of disease-specific EV populations.

In this work we have characterized three distinct nanobody clones, each selected by panning a naïve VHH library directly on EV derived from the cell culture supernatant. All examined nanobodies have excellent physicochemical properties resulting in outstanding expression and solubility. Positive isoelectric points at alkaline pH and negative GRAVY scores indicate hydrophilic structures. Aliphatic indices, especially high ones in NA8, point towards robust thermal stability and a structurally stable back-bone.

The AlphaFold-predicted models of the three VHH domains (Figure 3A–C) demonstrate the characteristic immunoglobulin fold with conserved β-sheet frameworks, which is consistent with previous structural analyses of nanobodies [27,33]. Notably, the CDR loops, especially CDR3, show considerable structural diversity and differences in confidence of predicted structures, as corroborated by the color gradient representing regions of lower pLDDT scores in panels A and C. Such diversity is in line with the intrinsic flexibility and sequence variability typical for CDR3 loops in VHHs, which provide antigen specificity and binding diversity [55]. While AlphaFold reliably predicts the conserved framework regions with high confidence, predicted CDR3 conformations are of medium to lower confidence, reflecting challenges in accurate modeling of very flexible or longer loops when there are no homologous structural templates [33,56]. The presence of a helical turn in CDR3 in panel C shows that AlphaFold can capture some noncanonical loop conformations, though such predictions would have to be experimentally confirmed or computationally refined [56]. Cumulatively, these models provide valuable structural insight into VHH diversity and can serve as a scaffold to inform antigen-binding research and engineering efforts, with a caveat that loop flexibility of the CDRs can limit prediction accuracy in some cases.

Each clone could successfully capture EVs from human plasma upon immobilization onto a solid carrier. In comparison to ultracentrifugation, similar number of vesicles with less proteins were secluded [57]. Particle size and concentrations were measured by NTA. The determined diameters of isolated EVs ranged from 50 to 300 nm with median diameters that correlate to those already reported in the literature [58]. In comparison to the Size-exclusion Chromatography (SEC) method reported by Gámez Valero et al. where 2 mL of plasma yielded 2.8 × 10^10^ particles, our nanobody-based capture system produced similar yields using a lower plasma input of 400 μL. Notably, despite the lower starting plasma volume, our best-performing nanobody (NA8) yielded comparable EV number when normalized to starting plasma volume, demonstrating high capture efficiency of our method [59]. SEC is generally a gentle method that preserves vesicle integrity and downstream analyses; however, it can involve sample dilution which requires further EV concentration [60] and therefore implies longer processing time. On contrary, compared to our nanobody-based approach, precipitation-based methods provide EV yields far exceeding ours, but highly contaminated, therefore often compromising downstream analysis reliability [59]. In addition, the precipitating agent can often remain in the EVs pellet and alter EVs function [61]. Immunoaffinity approach has been shown to provide a balance between yield, purity and usability. Compared to antibodies, VHHs can offer superior immunoaffinity EVs purification by enabling denser immobilization due to their size, exhibiting higher stability and improved epitope accessibility on vesicles. Unlike conventional methods, such as ultracentrifugation, SEC or precipitation-based methods, it can enable selective recovery of EVs subpopulations. The protocol is time-efficient, usually requiring less than two hours, it is easy to implement and does not require any special instrumentation. In terms of costs and functionalization density, the small mass of VHHs represents a critical advantage with respect to the bulky conventional IgGs [62]. In our previous work [31] we have employed a polyclonal cocktail of five VHHs from a naïve library for EV capture. Binding kinetics were not determined, but we assumed that inherent affinities were likely of moderate magnitude, and this was optimal for vesicle mild and efficient release. The method provided EV preps from plasma containing ~0.11 mg protein/mL and 3.94 mg lipids/mL and 2.3 × 10^9^ particles/mL of plasma. In this work, we compared three single VHHs (NA8, ND10_2_, ND10_1_) for their ability to capture EVs. As previously, absolute affinities were not assessed, although relative recovery efficiency indicated that NA8 consistently outperformed ND10_2_ and ND10_1_. From 0.4 mL plasma, NA8 recovered 4.57 × 10^9^ particles (equivalent to ~1.1 × 10^10^ particles/mL plasma), while ND10_2_ and ND10_1_ recovered 2.37 × 10^9^ (~5.9 × 10^9^/mL) and 1.27 × 10^9^ (~3.2 × 10^9^/mL), respectively. The protein recovery was between 0.89 and 1.07 mg/mL plasma and lipid were between 0.25 and 0.38 mg/mL plasma depending on the VHH clone. Together, the current study extends our previous work by highlighting clone-specific differences in capture efficiency and showing that individual VHHs can achieve plasma EV yields comparable to those obtained by established isolation methods [31]. Importantly, although the vesicles were eluted using low-pH buffer, their spherical structure and morphology remained intact, as confirmed by SEM and AFM analyses. Canonical EV surface markers CD9, CD63, and CD81, have been confirmed using flow cytometry on NA8, ND10_1_, and ND10_2_ plasma-derived EVs. Decrease in fluorescence signal after addition of Triton X-100 confirmed the presence of vesicular structures and absence of protein aggregates in all three isolates. These results verify the integrity and biochemical homogeneity of the EV samples, making them eligible for downstream functional and molecular analyses.

In our study, the isolated vesicles were also characterized by ATR-FTIR. Due to its simplicity, and minimal sample requirements, ATR-FTIR spectroscopy is recognized as a powerful tool for analyses of biological samples. ATR-FTIR is a non-destructive, stable technique for EV characterization. Moreover, this technique has previously been used for characterization of EVs, including comparison of isolation performances [63], quantification of protein content in isolates [64], classification of EVs and determination of protein-to-lipid ratio [65], as well as the assessment of their potential application in cancer diagnostics [66,67]. The amide I, amide II, and amide A bands originating from EV proteins, as well as bands from C-H stretching vibrations of CH_2_/CH_3_ from lipids are clearly distinguishable in the ATR-FTIR spectra [63,65]. These results indicate that the vesicles captured with the nanobody-based approach retained their expected molecular composition. According to Mihály et al. [65] IR spectra of EVs show a higher relative amount of the lipid component comparing to protein, which correlates with our obtained P/L ratios further confirming presence of extracellular vesicles in our samples. FTIR-obtained P/L ratios of the three samples under the study are well below the commonly used cut-off ratio of 1.0 indicating high-purity EV preparations with minimal contamination by protein aggregates or lipoproteins [68]. These low ratios are consistent with the structure of isolated vesicles which is lipid bilayer-rich and differs from that of protein. They also show that the employed isolation method was effective at minimizing co-isolated non-EV protein. This analysis is consistent with recent studies demonstrating the utility of FTIR lipid-to-protein ratio analysis as a reliable and operator-independent EV quality control approach [68]. The difference between P/L ratios obtained from the colorimetric measurement and ATR-FTIR data could be a result of inability of colorimetric dyes to access interior of cargo proteins because of the unusual structure of EVs (stabilized spherical vesicles). When comparing the results from the various techniques, the highest protein concentration was determined from data obtained by the ATR-FTIR. The effectiveness of the dye–protein interaction does not affect IR spectroscopy since it is a label-free approach. The IR beam passes through an internal reflection element (ATR crystal) in the ATR-FTIR mode and reflects from the internal surface of the crystal which is in contact with the sample. As a result of reflection, an evanescent wave emerges, penetrates the sample, and is absorbed by the sample. The absorbance is translated into the IR spectrum. The entire protein content of EVs samples was analyzed and assessed because the penetration depth of this evanescence wave normally varies between 1 and 2 μm within the 1800–900 cm^−1^ region [64].

Although this work demonstrates the successful use of three different nanobodies for the isolation of EVs, an important question still remains open regarding the precise identity and EV subpopulations captured by each nanobody. Even though the recovered vesicles exhibited similar physicochemical and biochemical properties, it is still unclear whether these nanobodies bind the same or distinct EV subpopulations. If the nanobodies indeed target different antigens, this may imply that they capture specific EV subpopulation, each potentially having unique functional role. Given the fact that isolated vesicles were confirmed to express canonical tetraspanin markers (CD9, CD63, CD81), but not at uniformly high levels across all isolates, it is plausible that the nanobodies bind epitopes distinct from commonly targeted tetraspanins.

Such divergence in antigen recognition would suggest that selected nanobodies could recognize some less-characterized EVs surface molecules, opening possibilities for the capture of new EV populations. However, the identification of nanobody targets remains unresolved and requires further investigation, such as epitope mapping. Therefore, future work should aim to evaluate the binding specificities of each nanobody and characterize the potential functional and molecular differences among the captured EV subtypes. Furthermore, thorough characterization on EVs populations targeted by these VHHs should be investigated by single vesicle analysis and omics methodologies. Addressing these questions would be important for advancing nanobody-based EV isolation to a precisely targeted and EV subpopulation specific isolation platform.

## 4. Materials and Methods

### 4.1. Chemicals

Molecular weight markers for SDS-PAGE were Pierce^TM^ Unstained Protein MW Marker, Thermo Fisher Scientific (Waltham, MA, USA). Antibodies used for flow cytometry were supplied from Biolegend (San Diego, CA, USA): anti-CD-9 Phytoerythrin (PE)-labeled (clone MM2/57), Alexa Fluor^®^ 488 anti-human CD63 (clone H5C6) and PE/Dazzle™ 594 anti-human CD81 (TAPA-1) antibody (clone 5A6). Aldehyde/sulfate latex beads used for flow cytometry were 4% *w*/*v*, 4 µm; Sigma-Aldrich (St. Louis, MO, USA). All other chemicals were of analytical grade.

### 4.2. Panning on Isolated EVs

Panning was carried out using a modified approach based on previously established methods with some modifications [30]. EVs for panning have been obtained by immunoaffinity isolation [31]; EVs were stabilized on epoxy magnetic beads (Life Technologies, Thermo-Fisher Scientific, Waltham, MA, USA) by means of anti-EVs-VHH-GFP (H1; 10 µg/25 µL of beads or per MPT well). Nanobody-displaying phages (3 × 10^11^) [50] were initially diluted in 1 mL of PBS with 2% skimmed milk and then depleted by incubating them twice for 30 min with 25 µL of milk-blocked anti-EVs-VHH beads, after which the bound fraction was discarded each time. The remaining phage fraction was transferred to the VHH stabilized HEK293 EV-coated beads and incubated for 1 h. This depletion process was repeated, and the final unbound phage fraction was incubated with beads coated with the EV-enriched fraction obtained from the immunoaffinity separation of SKBR3 culture media., as described above. The pre-selected phages were then incubated with the EV-coated beads for 1 h at room temperature, and the unbound fraction was discarded. Following 20 washing cycles in PBS, the bound phages were eluted by adding 1 mL of 200 mM glycine at pH 2.2 containing 1 mg/mL BSA and then neutralized with 150 µL of 1 M Tris–HCl at pH 9.1. The phages were subsequently used to infect TG1 cells spread over 2xTY Petri dishes. The resulting colonies were collected the following day, and the corresponding rescued phages were utilized for a second panning cycle.

### 4.3. Selection of Enriched Phage Libraries

The colonies that were obtained after the second panning cycle generated phages, which were then evaluated by ELISA for their ability to bind to EVs. A non-related clone served as a negative control. Microtiter plates (96-well, MaxiSorp, Nunc™, Roskilde, Denmark) were coated by incubating at 4 °C overnight with 10 μg/well in PBS of anti-EVs-VHH. Plates were sealed (1 h at 37 °C in PBS buffer with 5% (*w*/*v*) skim milk) after which immunoaffinity isolated EVs form HEK and SKBR3 cell cultures were added in concentration of 0.1 µg/well. Phages were pre-treated for 1 h at 37 °C in PBS buffer with 1% (*w*/*v*) skim milk before addition to the plates. One hundred microliters of blocked phages were added to each well of the plates and incubated for 1 h at room temperature. Subsequent to washing (4 times × 5 min in PBS), each well received 100 μL of mouse anti-M13 antibodies conjugated with HRP (1:5000 dilution, GE Healthcare, Chicago, IL, USA) and was incubated for 1 h at 37 °C. Following an additional washing step (4 times × 5 min in PBS), 100 µL of TMB solution (Sigma Aldrich, St. Louis, MO, USA) was added to generate the reporter signal. The color reaction was halted by 50 μL of 2 NaHSO_4_. Absorbance at 405 nm was measured with an HTS7000 Bioassay reader (Perkin Elmer, Waltham, MA, USA).

### 4.4. Physicochemical Analysis of VHH Nanobodies

Physicochemical properties were calculated using the Biopython ProtParam module. Molecular weight, isoelectric point (pI), instability index, aromaticity, hydrophobicity (GRAVY), and aliphatic index of each nanobody sequence were determined. GRAVY was measured as average hydropathy values, while aliphatic index was calculated manually from relative volume of aliphatic side chains (Ala, Val, Ile, Leu).

### 4.5. Comparison of Nanobody Sequences and Structures to Canonical VHH Framework

Protein sequences of NA8, ND10_1_, and ND10_2_ variants were not aligned and varied in length. Pairwise global alignments were performed using the Biopython pairwise2 module with the default values for examining sequence conservation and variation. NA8 was used as a reference sequence to align against ND10_1_ and ND10_2_ and to achieve pairwise alignments. These were then padded to equal length to create a multiple sequence alignment (MSA) for visualization.

To determine the degree of conservation and variability in VHHs, three nanobodies—NA8, ND10_1_, and ND10_2_—were aligned with a canonical llama VHH germline sequence, CabBCII-10. Several primary sequence alignments were created using the Biopython PairwiseAligner 1.86.dev0 and the BLOSUM62 substitution matrix. IMGT numbering schemes were used to approximate complementarity-determining regions (CDRs).

VHH structures were predicted using AlphaFold2 (v) via the open-source implementation provided by DeepMind: https://github.com/deepmind/alphafold (accessed on 23 July 2025). Predictions were visualized and analyzed using PyMOL and confidence metrics (pLDDT) provided by AlphaFold. AlphaFold models were further analyzed using MolProbity [34]. CDR loops were identified by IMGT numbering: CDR1 (residues 27–38), CDR2 (56–65), and CDR3 (105–116). [69] CDR sequences were aligned to identify sequence variability, and structural models were visually inspected to identify loop conformations, framework conservation, and paratope architecture using IMGT DomainGapAlign tool: https://www.imgt.org/3Dstructure-DB/cgi/DomainGapAlign.cgi (accessed on 23 July 2025) [70,71].

### 4.6. Expression and Purification of VHH-eGFP Constructs

In this work three different VHH, labeled as NA8, ND10_1_, ND10_2_ were used and isolated from a naïve-preimmune library by direct panning on EVs. Plasmids containing VHH genes were transformed into *E. coli* BL21(DE3) competent cells with expressed sulfhydryl oxidase and DsbC and plated on agar with selective medium. One milliliter of pre-culture was transferred into 200 mL of LB broth containing antibiotics (100 μg/mL ampicillin, 25 μg/mL chloramphenicol) and bacteria were grown at 37 °C, 220 rpm until the OD_600_ reached 0.4. L-arabinose was added to a final concentration of 0.2% (*w*/*v*) for expression of sulfhydryl oxidase and DsbC, and the temperature was lowered to 30 °C for 30 min. To induce nanobody expression IPTG (0.2 mM final) was added. Bacteria were incubated for 12 h at 20 °C. Subsequently, cells were pelleted and resuspended in 20 mL of resuspension buffer (500 mM NaCl, 5 mM MgCl_2_, 50 mM Tris-HCl, pH = 7.4), with the addition of lysozyme (0.5 mg/mL final) and DNAse (1 U final). After incubation for 30 min at RT, cells were sonicated and centrifuged at 11,000× *g* for 15 min. Nanobodies from the supernatant were purified using IMAC. All used buffers had a pH value of 7.4. The column was equilibrated with wash buffer (50 mM Tris-HCl, 500 mM NaCl, 30 mM imidazole). The unbound fraction of proteins was removed in two steps: first using wash buffer and second using wash buffer 2 (50 mM Tris-HCl, 500 mM NaCl, 100 mM imidazole). VHH-eGFP constructs were then eluted with elution buffer (50 mM Tris-HCl, 500 mM NaCl, 300 mM imidazole). Ten% glycerol was added to the purified nanobodies for long-term storage at −20 °C.

### 4.7. Coupling of Nanobodies to a Solid Carrier

Methacrylate-based copolymer was used for immobilization of nanobodies. Activation and immobilization of the polymer were performed as previously described with some minor modifications [31]. Nanobodies were attached to the polymer by incubating 500 μL of wet polymer with 100 μg of each VHH-eGFP construct. A Bradford assay was used to measure protein concentration before and after coupling to determine the efficacy of immobilization. The immobilized nanobodies were rinsed 3 times with 0.1 M sodium-phosphate buffer pH 7. Free binding sites were blocked with 200 mM glycine (pH 7) for 30 min at RT with shaking. Additionally, binding sites were subsequently blocked with 5% (*w*/*v*) skimmed milk in PBS under the same conditions. Prepared immobilization system was equilibrated with PBS and used for purification of EVs from human plasma.

### 4.8. Purification of EVs from Human Plasma Using Immunoaffinity Approach

Human blood was collected from one healthy donor using a citrate tube. The study was approved by the Ethics Committee of University of Belgrade, Faculty of Chemistry (2-6/24, 16 July 2024). The collection was performed in accordance with the Declaration of Helsinki. Blood was processed 20 min after collecting, and plasma was isolated by previously established protocol [31]. Immobilized polymer (0.5 mL) was incubated with 1.2 mL of 3 × diluted plasma with PBS for 1 h at RT with light shaking. Unbound fractions were washed with PBS until a negative Bradford spot test. Bound EVs were eluted in batch by incubating 400 μL of 200 mM glycine pH 2.0 and polymer carrier for 15 min with light shaking. The eluted fraction was collected into a tube with 100 μL of 1 M Tris-HCl buffer (pH = 9) for neutralization. This elution step was repeated, and two batches of eluted EVs were pooled. Prior to further analyses, EV samples were dialyzed against 0.1 μm filtered PBS.

### 4.9. SDS PAGE Analysis of Purified Nanobodies and EVs Isolates

The purity of the purified recombinant nanobodies and the protein profile of the isolated EVs were analyzed by Sodium Dodecyl Sulfate-Poly Acrylamide Gel Electrophoresis (SDS-PAGE) according to Laemmli [72] in 12% polyacrylamide gels. After SDS-PAGE, protein bands were visualized using Coomassie Brilliant Blue. Stained gels were visualized and analyzed using Gel Analyzer 23.1.1: available at www.gelanalyzer.com (accessed on 15 July 2025) by Istvan Lazar Jr., PhD and Istvan Lazar Sr., PhD, CSc.

### 4.10. Determination of Protein and Lipid Content in EVs Isolates

Protein concentration in EV isolates was determined with the Bradford assay [73] by mixing 200 μL of work reagent with 20 μL of sample, using BSA as a standard. Lipid concentration in EV isolates was determined using the sulfo-phospho-vanillin (SPV) assay as previously reported [74] and expressed based on the cholesterol content. Standards were prepared by dissolving cholesterol in chloroform in concentrations ranging from 0.02 to 0.250 mg/mL.

### 4.11. Quantification and Size Distribution of EVs by NTA

The size distribution and median size of the EV isolates were assessed using the ZetaView Quatt PMX-430 (Particle Metrix, Inning am Ammersee, Germany) nanoparticle tracking analyzer and ZetaView software version 8.05.16. Prior to analysis, the camera and laser underwent an automatic cell check, with focus confirmed using 100 nm polystyrene beads as described in the manufacturer’s guidelines. EV samples were diluted in particle-depleted 0.05 M PBS, pH 7.2 (dPBS) to achieve an optimal particle count per frame. Measurements were conducted using a blue laser (488 nm) in light scatter mode (LSM), with dPBS used for washing between each measurement. Video acquisition settings included a shutter speed of 100 and a frame rate of 30 per cycle, with sensitivity set at 78. Post video-capturing parameters included a minimum area of 10, a maximum area of 1000, and a minimum brightness of 30. Each EVs isolate was measured three times at up to 11 positions.

### 4.12. Attenuated Total Reflection Fourier-Transform Infrared Spectroscopy (ATR-FTIR)

ATR-FTIR spectra were collected using Nicolet Summit FTIR Spectrometer (Thermo Fisher Scientific, Waltham, MA, USA) in ATR mode equipped with DTGS KBr detector (Thermo Scientific, Waltham, MA, USA). Aliquots of 1.5 μL of EVs isolates were applied onto a diamond crystal and a thin dry film was obtained by evaporating the buffer in the stream of nitrogen. Spectra (400–4000 cm^−1^) were collected in 128 scans with a resolution of 4 cm^−1^. OMNIC 7.3 software was used for automated ATR correction, while Spectragryph 1.2 software was employed for integration of areas under amide I and lipid bands.

### 4.13. Analysis of Surface EVs Markers

Latex beads were diluted 10 times in PBS. A total weight of 20 μg of EVs (based on protein concentration) was immobilized onto 40 μL of diluted beads overnight at 4 °C with light shaking. The following day the beads were washed three times with PBS (the beads were vortexed, then pelleted at 13,000× *g*, 5 min) and initially blocked with 200 mM glycine (pH 7.0) for 30 min at RT with light mixing, followed by 5% (*w*/*v*) skimmed milk under the same conditions. The milk was removed and the beads were washed with PBS until no milk residues were left. Antibodies were diluted 5 times in PBS with 1% skimmed milk and incubated with the beads for 1 h at RT with light shaking. After washing the excess antibodies with PBS, prepared samples were analyzed with BD FACS Calibur (BD Biosciences, San Jose, CA, USA), using blue state laser (488 nm) for excitation. The emissions were detected at following wavelengths: 525 nm (Alexa fluor 488), 561 nm (PE) and 620 nm (PE/Dazzle). The positive shifts in fluorescence were measured against beads blocked with milk that were not coated with EVs for control. For negative control, Triton X-100 solution (1%) was added to the samples to final concentration of 0.5% (*v*/*v*) and incubated for 30 min before measuring.

### 4.14. Evaluation of EVs Morphology

#### 4.14.1. Scanning Electron Microscopy (SEM)

The morphology of isolated EVs was analyzed by scanning electron microscopy (SEM). Isolates were fixed with 2.5% glutaraldehyde in PBS for 10 min at RT, further loaded on metal stubs, and left to dry. All samples were coated with a thin-gold layer using a sputter coater (Polaron SC503, Fisons Instruments, Ipswich, UK) prior to characterization of the EV morphology with Tescan FE-SEM Mira 3 XMU (Tescan, Brno, Czech Republic)

#### 4.14.2. Atomic Force Microscopy

The surface morphology of isolated EVs was examined with atomic force microscopy (AFM) using a NanoScope 3D (Veeco, NY, USA) microscope in tapping mode under ambient conditions. Image analysis was conducted utilizing Nanoscope image processing software (v1.40r1). Prior to imaging, the mica substrate was mechanically polished using adhesive tape. Ten μL of EV isolates were applied to prepared mica substrate and allowed to air-dry for 7 days.

### 4.15. Statistical Analysis

Statistical analysis was performed using GraphPad Prism 10.4.2. for Windows (San Diego, CA, USA). A significance level of *p* ≤ 0.05 was used for analysis of variance, implemented using the one-way ANOVA test followed by Tukey’s post hoc test (*p* ≤ 0.05). Values with significance level of *p* ≤ 0.05 were considered statistically different.

## 5. Conclusions

In this study, successful isolation and characterization of plasma-derived EVs using three different nanobodies selected by direct panning on EVs have been demonstrated. This nanobody-based immunoaffinity approach is fast, easy-to-operate, and ensures high recovery of intact EVs from human plasma. Importantly, nanobodies offer many advantages over conventional antibodies, which makes them powerful tool for development of selective, reproducible and scalable EVs isolation strategies. Our findings support the application of nanobodies for immunoaffinity-based methods as an effective alternative to conventional antibody-based approaches, with strong potential for diagnostics and research applications.

## Figures and Tables

**Table 1:** Sequences of CDR Loops of NA8, ND10_1_, and ND10_2_ nanobodies.

Nanobody	CDR1	CDR2	CDR3
**NA8**	GVTLGNYV	YRRSHGNK	ALTMSRPILSSLPDRSIYDY
**ND10_1_**	GRTFSV	ISRTLGRT	AVRSSGFYGQVPRVGESYDY
**ND10_2_**	GRTSDIYR	ITSGGST	AADRHYSTNYYSERVYDY

**Table 2:** NTA of EV isolated with three different nanobodies.

	Median Diameter (nm)	Total Yield (Particles)
**NA8-EVs**	138.1 ± 2	4.57 × 10^9^
**ND10_1_-EVs**	137.3 ± 3	1.27 × 10^9^
**ND10_2_-EVs**	134.6 ± 1	2.37 × 10^9^

**Table 3:** Quantification of total proteins and lipids in EVs isolates. Values with different letters are significantly different.

	Protein Yield (mg)	Lipid Yield (mg)	P/L Ratio
**NA8-EVs**	0.357 ± 0.010 ^a^	0.132 ± 0.006 ^d^	2.740 ± 0.074 ^f^
**ND10_1_-EVs**	0.429 ± 0.007 ^b^	0.101 ± 0.031 ^d^	4.248 ± 0.323 ^g^
**ND10_2_-EVs**	0.388 ± 0.011 ^c^	0.153 ± 0.019 ^d^	2.536 ± 0.153 ^f^

## Data Availability

Data can be found within the article and Supplementary Materials.

## Supplementary Materials

The following supporting information can be downloaded at: https://www.mdpi.com/article/10.3390/molecules30183677/s1, Figure S1: Densitometric analysis of isolated EVs using: (A) NA8; B) ND10_1_ and (C) ND10_2_. Rf values and band intensities are nearly identical with all 3 VHH indicating highly similar sample composition.; Table S1: Physicochemical properties of VHH nanobodies.

## Abbreviations

The following abbreviations are used in this manuscript: AFM—Atomic Force Microscopy; ATR-FTIR—Attenuated Total Reflectance-Fourier Transform Infrared Spectroscopy; CDR—Complementarity-Determining Regions; EVs—Extracellular Vesicles; EXO—Exosomes; FR—Framework Region; IMAC—Immobilized Metal Affinity Chromatography; MVB—Multivesicular Bodies; NTA—Nanoparticle Tracking Analysis; SEC—Size-Exclusion Chromatography; SEM—Scanning Electron Microscopy; VHH—Single-Domain Antibodies/Nanobodies
