# Supplementary material for: Implementation of a Novel Nanobody Panel for the Efficient Capture of Extracellular Vesicles from Human Plasma
Source: Molecules. 2025 Sep 10;30(18):3677. doi: 10.3390/molecules30183677 (PMC12472977; doi:10.3390/molecules30183677)
Supplement: Supplementary file 1 [file molecules-30-03677-s001.zip › molecules-3817939-supplementary.pdf]

Nanobody	Length	MW (Da)	Instability Index	pI	Aromaticity	GRAVY	Aliphatic Index
**NA8**	127	14039.60	46.83	9.20	0.110	-0.393	73.62
**ND10-1**	125	13491.91	46.32	9.30	0.112	-0.241	69.44
**ND10-2**	125	13955.18	39.79	8.59	0.136	-0.632	55.52

Table S1. Physicochemical properties of VHH nanobodies.

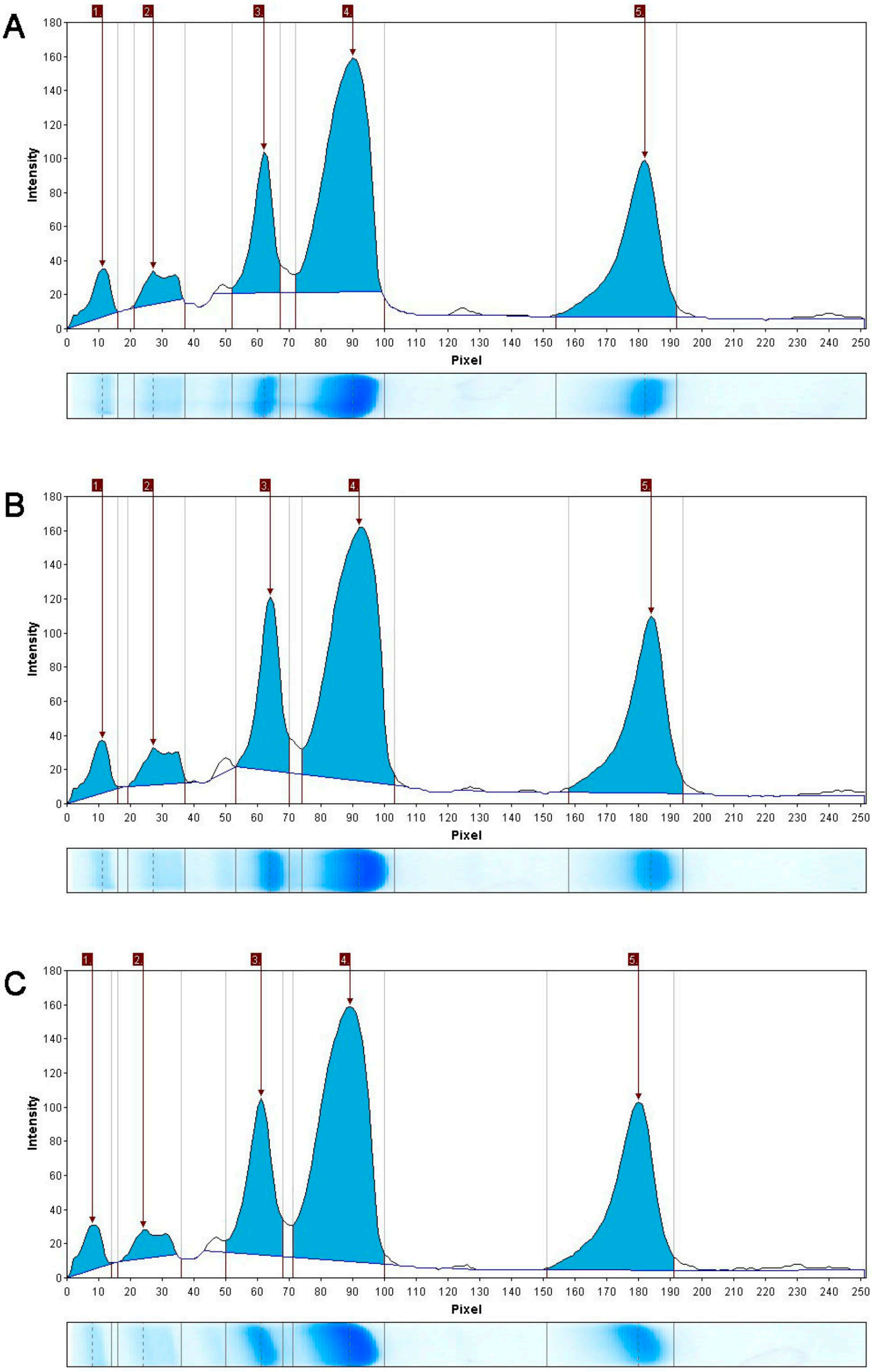

Figure S1. Densitometric analysis of isolated EVs using: A) NA8; B) $ND10_1$ and C) $ND10_2$. Rf values and band intensities are nearly identical with all 3 VHH indicating highly similar sample composition.
